# N^6^-Methyladenosine in Cancer Immunotherapy: An Undervalued Therapeutic Target

**DOI:** 10.3389/fimmu.2021.697026

**Published:** 2021-08-30

**Authors:** Chao Quan, Othmane Belaydi, Jiao Hu, Huihuang Li, Anze Yu, Peihua Liu, Zhenglin Yi, Dongxu Qiu, Wenbiao Ren, Hongzhi Ma, Guanghui Gong, Zhenyu Ou, Minfeng Chen, Yin Sun, Jinbo Chen, Xiongbing Zu

**Affiliations:** ^1^Department of Urology, Xiangya Hospital, Central South University, Changsha, China; ^2^Center for Inflammation and Epigenetics, Houston Methodist Research Institute, Houston, TX, United States; ^3^George Whipple Lab for Cancer Research, University of Rochester Medical Center, Rochester, NY, United States; ^4^Department of Radiation Oncology, Hunan Cancer Hospital, Central South University, Changsha, China; ^5^Department of Pathology, Xiangya Hospital, Central South University, Changsha, China

**Keywords:** N^6^-methyladenosine, m^6^A, tumor microenvironment, immune response, immunotherapy

## Abstract

N^6^-methylation of adenosine (m^6^A), a post-transcriptional regulatory mechanism, is the most abundant nucleotide modification in almost all types of RNAs. The biological function of m^6^A in regulating the expression of oncogenes or tumor suppressor genes has been widely investigated in various cancers. However, recent studies have addressed a new role of m^6^A modification in the anti-tumor immune response. By modulating the fate of targeted RNA, m^6^A affects tumor-associated immune cell activation and infiltration in the tumor microenvironment (TME). In addition, m^6^A-targeting is found to affect the efficacy of classical immunotherapy, which makes m^6^A a potential target for immunotherapy. Although m^6^A modification together with its regulators may play the exact opposite role in different tumor types, targeting m^6^A regulators has been shown to have wide implications in several cancers. In this review, we discussed the link between m^6^A modification and tumor with an emphasis on the importance of m^6^A in anti-tumor immune response and immunotherapy.

## Background

The expression of genetic information is regulated at multiple levels. RNA modification, a post-transcriptional regulatory mechanism, is thought to alter the RNA function through changing the chemical-structural features of RNA (1). One hundred and seventy-two kinds of RNA modifications have been reported, most of which are restricted to non-coding RNA (ncRNA) instead of messenger RNA (mRNA) (2, 3). Unlike most other RNA modifications, m^6^A, which is first identified in the 1970s (4, 5), is an abundant nucleotide modification in almost all types of RNAs, including mRNA and ncRNA, and is involved in various physiological and pathological processes (6). Generally, the rise in m^6^A methylation in cancer cells plays a tumor-suppressor role with only a couple of studies showing the opposite (7–9). By targeting specific RNA, m^6^A triggers certain alterations to tumor-specific mRNA/ncRNA behavior and biologic activity, thereby influencing tumor progression.

Compared with the conventional therapies (radiation and chemotherapy) for cancers that directly target tumor cells, anti-tumor immunotherapy, which promotes a systemic immune response against tumors, exhibits great potential in cancer treatment (10). m^6^A, whose intrinsic function in tumor cells has been widely studied and discussed, is proved to influence the anti-tumor immune response and can be a novel target in anti-tumor immunotherapy (11–14). In this review, we briefly introduce the regulation and function of m^6^A, with an emphasis on the role of m^6^A in anti-tumor immune response and a summary of prominent m^6^A-related functions and strategies in immunotherapy.

## The Regulation and Function of m^6^A Modification

The m^6^A modification, a dynamic and reversible methyl-modification occurring at the N^6^ position of adenine (A) bases in RNA, is enriched in the internal exons, 3’ untranslated region (3’UTR) and the stop codon and restricted to a consensus sequence DRACH (D=G, A, or U; R=G or A; H=A, C, or U) (15–17). Generally, the regulation and function of m^6^A modification involve three kinds of protein factors: methyltransferases (writers), binding proteins (readers), and demethylases (erasers).

### Writers

‘Writers’ mediate m^6^A modifications by adding methylation to RNA. The classic ‘writer complex’ includes a core heterodimer consisted of methyltransferase-like 3 (METTL3) and methyltransferase-like 14 (METTL14), together with several accessory proteins (Wilms tumor 1-associated protein (WTAP), zinc finger CCCH domain-containing protein 13 (ZC3H13), RNA binding motif protein 15 (RBM15) or RBM15B and Vir like m^6^A methyltransferase associated (VIRMA) (11). METTL3 is responsible for catalyzing methyl-translocation from methyl donor [S-adenosylmethionine (SAM)] to RNA, while METTL14 mediates m^6^A deposition through recognizing and binding to the target RNA (18, 19). As a regulatory subunit, WTAP interacts with the METTL3/14 heterodimer and mediates the localization of the heterodimer in nuclear speckles (20). ZC3H13, RBM15 or RBM15B, and VIRMA are all WTAP-related and facilitate the nuclear localization and specificity of the ‘writer complex’ (21–23). In addition to the classic ‘writer complex’, several other enzymes independently catalyze the methyl transfer to target RNA, including METTL16 targeting U6 small nuclear RNAs, zinc finger CCHC-type containing 4 (ZCCHC4) targeting ribosomal RNAs, METTL5 targeting 18S ribosomal RNAs and PDX1 C-terminal inhibiting factor 1 (PCIF1) targeting 7-methyl guanosine (m7G)-capped mRNAs (11).

### Readers

‘Readers’ bind to m^6^A on modified RNAs and induce various biological effects (24). YT521-B homology (YTH) domain-containing ‘readers’, which bind RNA in an m^6^A-dependent manner, are categorized into three types: YTHDC1, YTHDC2 and YTHDF family proteins (YTHDFs) (11). YTHDC1 mainly affects mRNA splicing within the nucleus and participates in the nuclear export of mRNA (25, 26), whereas YTHDC2 promotes the translation as well as degradation of the targeted mRNA in/out the nucleus (27–29). There are three members in the YTHDF family: YTHDF 1, 2 and 3. However, their functions remain unclear, and further studies are needed (30). Some other RNA-binding proteins which preferentially bind to m^6^A-containing RNA are thought to be ‘readers’ too (11). However, more studies are also needed to decipher their regulatory functions and determine whether their preference for m^6^A-containing RNA is due to specific recognition for m^6^A (31).

### Erasers

‘Erasers’ are responsible for removing m^6^A from mRNA. The fat-mass and obesity-associated protein (FTO) and Alpha-ketoglutarate-dependent dioxygenase ALK B homologue 5 (ALKBH5) are two demethylases that proved to remove methylation on adenosine from RNA (17, 32, 33). FTO removes both m^6^A and N^6^,2′-O-dimethyladenosine (m^6^Am), whereas ALKBH5 specifically demethylates m^6^A (17, 32). Though FTO and ALKBH5 both are ‘erasers’, their biological function can be quite different. They act as either an oncogene or a tumor suppressor depending on tumor types (6, 34). It is clear that the different biological effect of ‘erasers’ is the result of demethylation of specific target mRNAs, but what leads to the mRNA selectivity still need to be addressed.

### Regulatory Function of m^6^A

**Figure 1:**
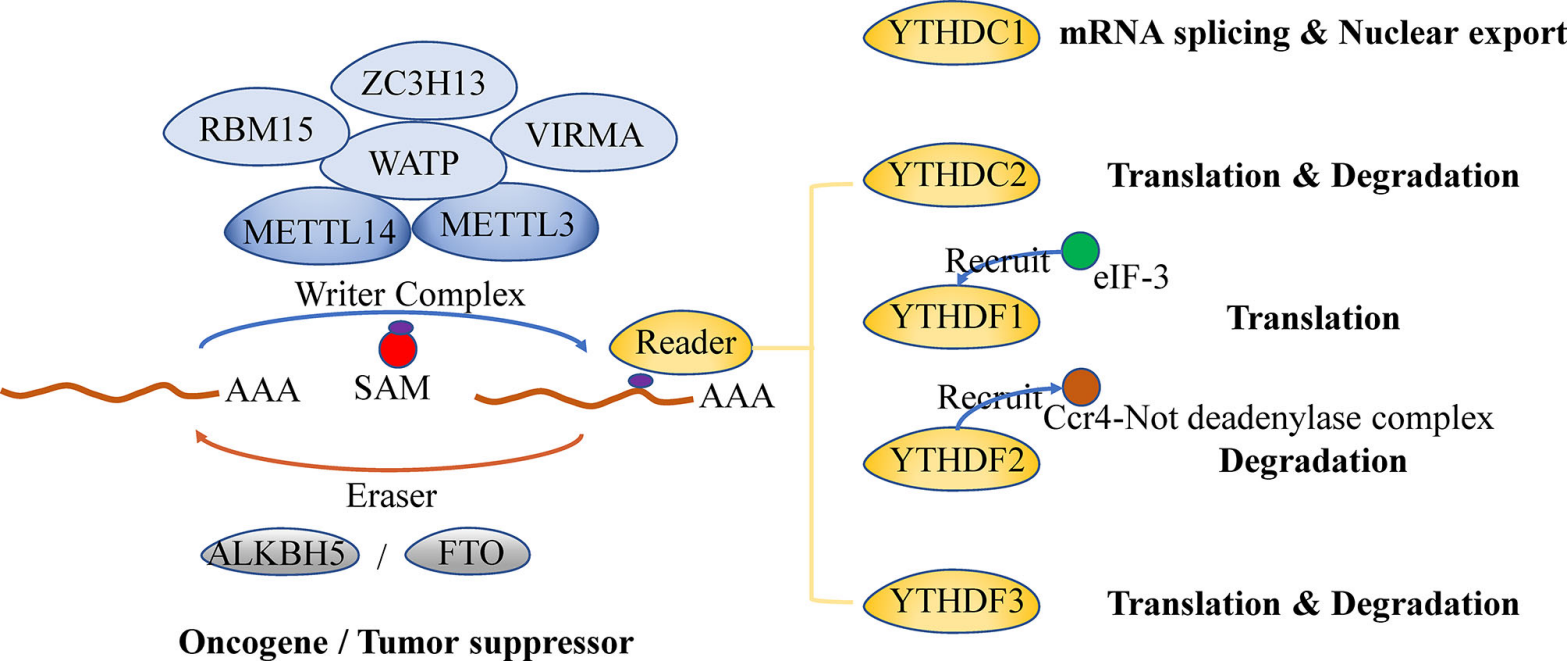
Regulator of m^6^A modification on mRNA and its biological functions.

The regulatory function of m^6^A on mRNA and ncRNA is very critical in a lot of key biological processes (**Figure 1**).

m^6^A is deposited on mRNA under the mediation of writers and then enrolls in the whole lifecycle of mRNA. Mediated by the readers, m^6^A affects mRNA splicing and export within the nucleus and influences the stability, translation and localization of mRNAs in the cytoplasm. It has been proved that m^6^A on precursor mRNAs (pre-mRNAs) promotes the mRNA splicing by directly recruiting splicing factor or indirectly altering mRNA structure and subsequently increasing the accessibility of flanking RNA sequence to splicing factors (35–38). Moreover, the m^6^A writers, readers, and erasers are all associated with mRNA splicing (17, 20, 25, 33, 39). The m^6^A modification also promotes mRNA export from the nucleus to the cytoplasm, which is mediated by writers or readers (40, 41). Although m^6^A modification is suggested to accelerate and promote the mRNA translation through several mechanisms, it still shows a negative correlation with mRNA half-life (42–44). This indicates that m^6^A may enhance mRNA translation by depending more on its readers rather than lengthening mRNA lifetime. A closed-loop model, in which eukaryotic translation initiation factor 3 (eIFs) subunit at the 5’ end of the mRNA interacts with METTL3 or YTHDF1 that is bound to m^6^A sites near the stop codon, is proposed to explain how m^6^A regulates mRNA translation (45, 46). YTHDFs, which specifically recognize m^6^A, are shown to regulate mRNA translation and degradation. YTHDF1 recruits eukaryotic translation initiation factor 3 (eIF-3) complex, which leads to enhanced mRNA translation (45), while YTHDF2 mediates the degradation of mRNA through recruiting Ccr4-Not deadenylase complex (42, 47). YTHDF3’s function is variable, which is linked with either mRNA translation promotion or degradation acceleration (48, 49). However, the insulin-like growth factor-2 mRNA-binding protein (IGF2BP) enhances the mRNA stability by recognizing m^6^A and facilitate mRNA translation (50–52). METTL3 promotes mRNA translation through directly recruiting eIF3 in the cytoplasm (46, 53). In addition, translational enhancement of m^6^A is partially mediated by the direct binding of eIF3 to m^6^A in the 5’ UTR (54).

In addition to mRNA, m^6^A and its regulators has also been found to affect the biological activity of other ncRNAs (55). METTL3-mediated m^6^A methylation on primary microRNA (pri-miRNA) are proved to promote the microRNA (miRNA) maturation in various pathophysiological processes (35, 56, 57). Mechanically, METTL3 methylates pri-miRNAs marking them for recognition and processing by DGCR8, which is the first step in the biogenesis of miRNAs (58). METTL3 may also promote the synthesis of mature miRNAs by increasing the splicing of pre-miRNAs by Dicer (59). As for long ncRNA (lncRNA), METTL3-mediated m^6^A modification on lung cancer associated transcript 3 (LCAT3), a long ncRNA (lncRNA), leads to the lncRNA stabilization (60), while METTL14-mediated m^6^A modification on lncRNA XIST facilitates the degradation of XIST which is attributable to YTHDF2 (61). In addition, m^6^A modification on circRNA has been identified and characterized (62). Furthermore, m^6^A modifications are believed to be involved in the biogenesis, translation, export and endoribonucleolytic cleavage of circRNAs, which has been discussed (63).

## Role of m^6^A in Anti-Tumor Immune Response

**Figure 2:**
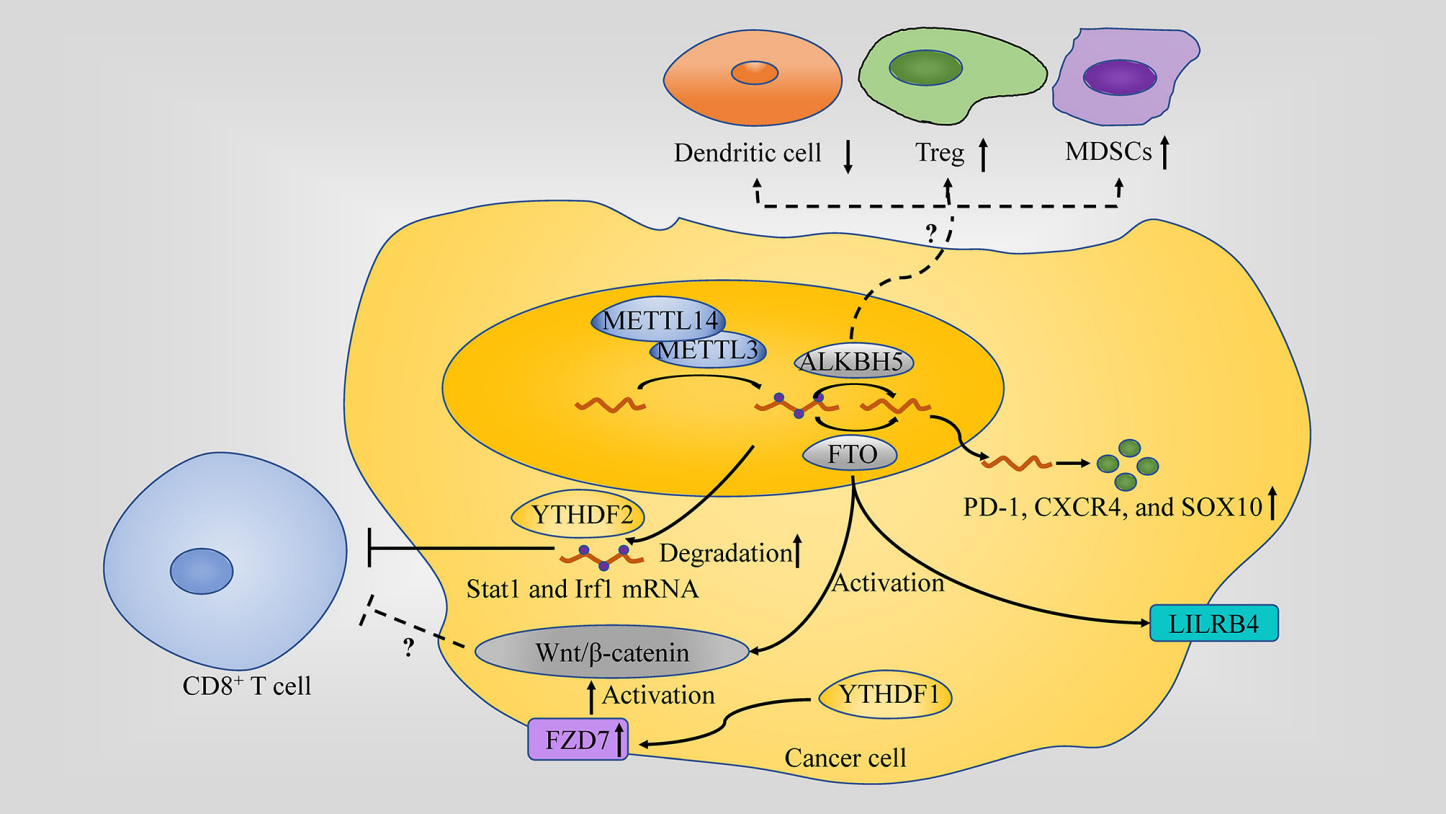
Role of m6A in the anti-tumor immune response. m^6^A regulators play different role in anti-tumor immune response: (1) METTL3 or METTL14 inhibit IFN-γ-Stat1-Irf1 signaling through decaying the Stat1 and Irf1 mRNA *via* Ythdf2. (2) FTO directly upregulate leukocyte Ig-like receptor B4 (LILRB4) which is an immune checkpoint gene expression *via* an m6A-dependent mechanism in AML; Through targeting PD-1, CXCR4, and SOX10FTO in cancer cell, FTO causes the resistance to the killing activity. (3) ALKBH5 affects the tumor infiltration of Tregs, MDSCs and DCs in TME. (4) Wnt/β-catenin hyperactivation through FZD7 overexpression which is mediated by YTHDF1 or by FTO is associated with non-T cell inflamed TME (a lack of CD8+ T cells along with DCs).

The role of intrinsic m^6^A modifications in modulating tumor fate through regulating specific target genes in different cancers has been thoroughly discussed (6, 62, 64). However, the link between aberrant m^6^A modifications and the anti-tumor immune response has received less attention, which has been indicated to be a potential therapeutic target in anti-tumor immunotherapy (**Figure 2**) (13, 65, 66).

### Role of m^6^A in TME Immune Response

The TME plays an important role in cancer progression and is also largely responsible for immunotherapeutic responsiveness. This is highlighted in tumor with T-cell-inflamed TME, containing abundant infiltration of CD8+ T cells (67), and thus predicting a better clinical outcome due to priming for the efficacy of immunotherapy (68), unlike the non-T-cell-inflamed TME. Interestingly, bioinformatical analysis shows that m^6^A modifications and its regulators are associated with the TME response and immune checkpoint blockade (ICB) efficacy in several tumor types. Moreover, it has been found that m^6^A modification influence the infiltration, activation and function of immune cell in TME, which makes m^6^A-targeting a promising therapy (65, 69).

#### m^6^A Is Associated With TME and Immunotherapy Efficacy

Many studies have confirmed the association between m^6^A modifications and TME condition in cancers with different histogenesis and organ location.

In renal clear-cell carcinoma (RCC), low m^6^A score group was characterized by a hot/inflammatory TME, which may be more sensitive to anti-tumor immunotherapy (70). Similar result was found in a RCC cohort that m^6^A score was negatively correlated with antigen processing machinery (APM) (71). Moreover, low m^6^A score, characterized by increased mutation burden and activation of immunity, indicates an inflamed TME phenotype and enhanced response to anti-PD-1/L1 immunotherapy in gastric cancer (GC) (65). In addition, m^6^A-related signature has been identified as biomarker for tumor immune phenotypes and anti-PD-1 immunotherapy treatment response in lung adenocarcinoma (LADC) (72, 73), stomach adenocarcinomas (STADs) (74), Esophageal squamous cell carcinoma (ESCC) (75, 76), Renal Papillary Cell Carcinoma (RPCC), Hepatocellular Carcinoma (HCC) (77, 78). These statistic results together indicate that m^6^A modification may reflect TME status and predict immunotherapy efficacy in pan-cancers instead of limited to specific cancer types. However, heterogeneity between these analysis limits the application in clinical practice, since each study focused on single cancer type and included different m^6^A indicator for analysis.

#### Remolding TME by Targeting m^6^A Regulator

Considering the potential effect of m^6^A on TME, it is promising to remolding TME through enhancing or reducing m^6^A modification by targeting m^6^A regulator.

METTL14, along with METTL3, affects the number of CD8+ T cells infiltrating into tumors since its depletion led to an increase of CD8+ T cells, in addition to as well as IFN‐γ, CXCL9 and CXCL10 in colorectal cancer (CRC) (13). Moreover, depletion of *Mettl3* and *Mettl14* enhanced the response to anti-PD-1 treatment in pMMR-MSI-L CRC and melanoma (13).

FTO, prompted by metabolic stress and starvation through the NF-κB signaling pathway and autophagy, increases melanoma growth and decreases response to anti-PD-1 blockade immunotherapy by protecting tumor cell-intrinsic genes (PD-1 (PDCD1), CXCR4 and SOX10) mRNA from decaying. In addition, in the case of endometrial cancer, FTO is also found to be changing the TME through regulating matrix metalloproteinase (MMPs), which are key player enzymes in promoting cancer growth (79, 80). Moreover, FTO can also directly upregulate leukocyte Ig-like receptor B4 (LILRB4) which is an immune checkpoint gene expression *via* an m^6^A-dependent mechanism in AML. CS1/CS2 (FTO inhibitor) treatment decreased the expression of LILRB4 in AML cells and substantially increased the sensitivity of AML cells to the cytotoxicity of activated T cells (81).

ALKBH5 alteration in tumor cell modulating its lactate content affects the tumor infiltration of Tregs and myeloid-derived suppressor cells (MDSCs) in TME, which are notorious immunosuppressants in anti-tumor immunity. ALKBH5 also regulates Mct4/Slc16a3 expression and DCs abundance. These regulations were highlighted when knocking down ALKBH5 along with administering GVAX/anti–PD-1 treatment resulted in Tregs and MDSCs infiltration of tumors to go down, DCs to increase and Mct4/Slc16a3 expression and lactate content in TME to decrease. ALKBH5, unlike FTO, is not changing the TME by affecting the IFN-γ pathway since the population of CD8+ T lymphocytes remains unchanged when the demethylase is depleted (82). In addition, single-cell mass cytometry analysis unveiled a role of ALKBH5 in TME by promoting the expression of PD-L1 on monocytes/macrophages and decreasing the infiltration of MDSCs (83).

In addition, Wnt/β-catenin pathway has been linked to m^6^A modifications and cancer in many studies. For example, Wnt/β-catenin signaling, which is, at least partially, overexpressed due to frizzled class receptor (FZD) 10’s m^6^A modification, contributes to epithelial ovarian cancers (EOC) resistance to poly ADP-ribose polymerase inhibitor (PARPi) (84). Wnt/β-catenin hyperactivation through FZD7 overexpression, a Wnt receptor, which has its elevation mediated by YTHDF1, leads to GC carcinogenesis (85) and Wnt pathway activation by FTO promotes endometrial cancer (86). Activation of the Wnt/β-catenin pathway is considered as a potential cause in developing the non-T cell inflamed TME since it is usually associated with poor T cell infiltration (a lack of CD8+ T cells along with DCs) among most human cancers. This activation also renders the tumors resistant to immune checkpoint therapy, in addition to resistance to vaccination and adoptive T cell transfer in the case of β-catenin-expressing melanomas. Therefore, targeting the Wnt/β-catenin pathway is a high priority for reversing non-T cell inflamed TME and sensitizing patients to different types of therapy (68).

### m^6^A in Immune Cell

**Figure 3:**
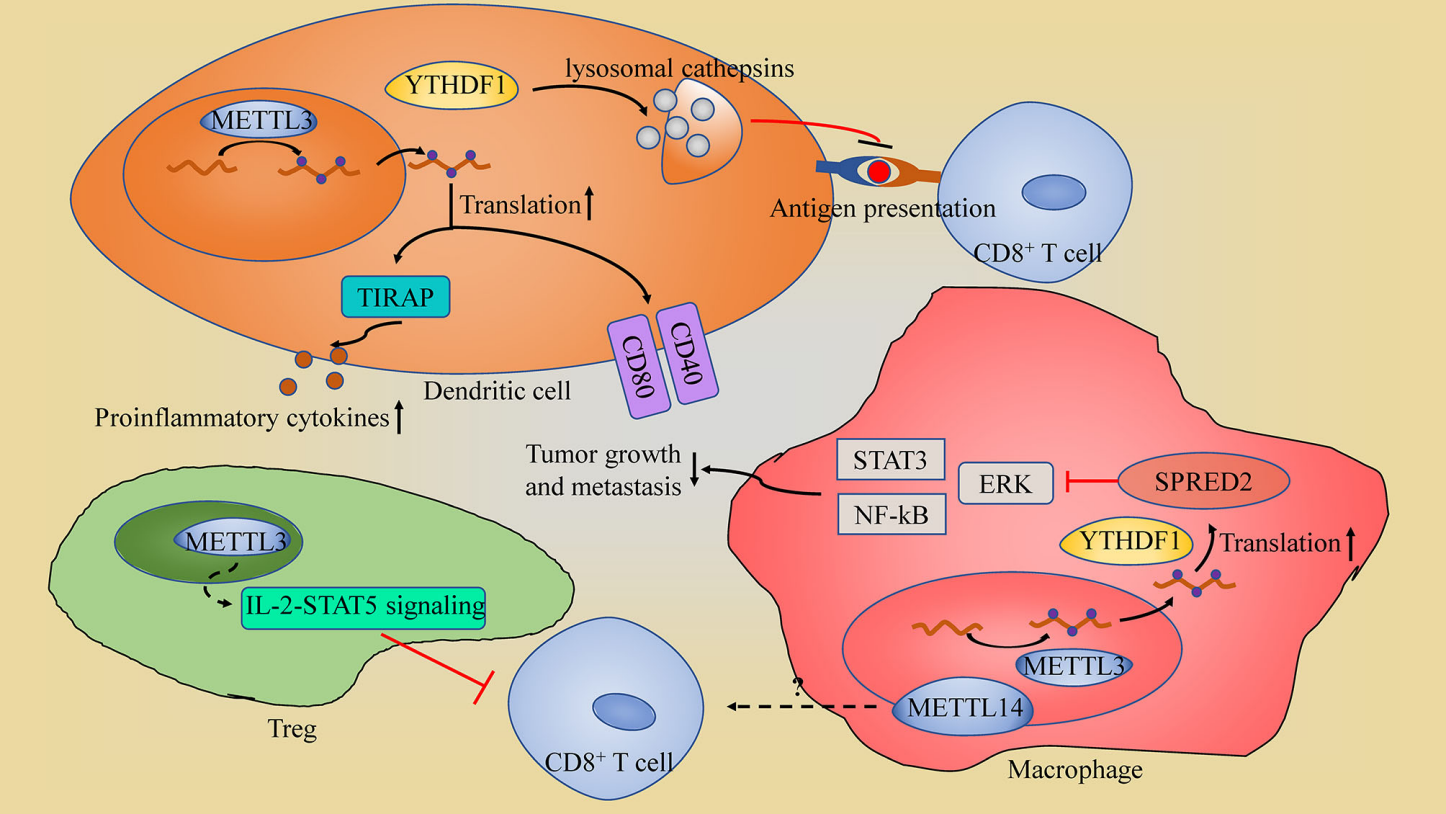
The schematic of m^6^A in immune cell. (1) In dendritic cells (DCs), YTHDF1 suppresses the antigen-presentation by DCs through maintain the expression of lysosomal cathepsins; METTL3-mediated m6A modification promotes DCs activation through enhancing the translation efficiency of CD40, CD80 and TIRAP. (2) METTL3 in Tregs sustains the suppression roles of regulatory T cells through IL-2-STAT5 signaling pathway impairing CD8+ T cells tumor killing ability. (3) In macrophages, METTL3 sustains the YTHDF1-mediated translation of SPRED2, which inhibits NF-kB and STAT3 through the ERK pathway, restricting tumor growth and metastasis; METTL14-deficiency in macrophage impairs CD8+ T cells to eliminate tumors.

As an important part of anti-tumor response, tumor infiltrating lymphocytes (TILs) and other immune cells is vital for shaping the TME and anti-tumor immunotherapy. While few published research on the effect of m^6^A on immune cell function is available (**Figure 3**).

METTL3-mediated m^6^A modification is found to be promoting DCs activation and maturation, leading to the induction of T cell activation. This promotion is through METTL3 enhancement of the translation efficiency of CD40, CD80 and TIR domain containing adaptor protein (TIRAP: a crucial TLR4/NF-κB signaling pathway adaptor, which induces proinflammatory cytokines secretion) (87). METTL3 depletion in macrophages contributed to the formation of an immunosuppressive microenvironment with increased Treg infiltration and reduced Th1 cells and IFN-γ+CD8+ cells, and subsequently facilitated tumor growth and metastasis (88). Moreover, METTL3-deficiency in macrophages impairs the YTHDF1-mediated translation of SPRED2, which enhances the activation of NF-kB and STAT3 through the ERK pathway, leading to increased tumor growth and metastasis (88). However, METTL3 also impairs CD8+ T cells tumor killing ability through sustaining the suppression roles of regulatory T cells (Tregs) (89). Depletion of *Mettl3* in Tregs suppresses the IL-2-STAT5 signaling pathway which is essential to Treg functions and stability (89). Thus, METTL3 in Tregs may suppress the anti-tumor immune response by maintaining the immunosuppressive function of Tregs. METTL14-mediated m^6^A modification in tumor-associated macrophages (TAMs) were found to modulate tumor-infiltrating CD8+ T cells, since macrophage-specific knockout of *Mettl14* drives CD8+ T cell differentiation along a dysfunctional trajectory, impairing CD8+ T cells to eliminate tumors (90).

YTHDF1 in DCs influences the anti-tumor immune response through impairing DCs’ lysosomal cathepsins. In summary, inhibition of YTHDF1 makes DCs more efficient at antigen-presentation and, at the same time, improves CD8+ T cross-priming; thus, giving them more ability to repress tumors than the non-lacking YTHDF1 cells (12). Moveover, YTHDF1 inhibition also increases PD-L1 checkpoint blockade potency in cancer regression (12).

To date, role of m^6^A modification in immune cells was given little attention and most studies was conducted in tumor model, with a few exceptions focus on other pathophysiological process, including autoimmune disease (91), T cell homeostasis and differentiation (92) and macrophage polarization (93). In addition, one single regulator may play the opposite role on immune response according to cell type, like METTL3 in DCs and Treg. Due to the inadequate data and confusing result, it’s hard to develop a strategy to promote anti-tumor immunity by targeting m^6^A in immune cell for now.

As discussed above, m^6^A modifications and its regulators affect tumor-associated immune cell activation and infiltration in TME. However, only a few m^6^A regulators has been investigated in several tumor or cell types. Moreover, m^6^A and its regulators play quite different role in the anti-tumor immune response according to tumor or cell type. Thus, more researches need to be done to identify the effect of aberrant m^6^A modification and exact biological function of each m^6^A regulators in different cancers or cell type context, especially in immune cells. In addition, m^6^A-targeting is found to affect the efficacy of classical immunotherapy preclinically, which further indicates the potential therapeutic value of m^6^A-targeting in tumor treatment.

## m^6^A Implication of m^6^A Modifications for Cancer Immunotherapy

Although m^6^A modification together with its regulators may play the exact opposite role in different types of tumors, targeting m^6^A regulators has been shown to have wide implications in several cancers. Since there is no clinical research investigating the efficacy of m^6^A-targeting strategy, we propose several pathways we might utilize to implement m^6^A area of research in the clinical settings of treatment, which is usually done by trying to selectively identify and then to inhibit the enzymes responsible for tumor exacerbation in specific cancers (**Table 1**).

**Table 1:** Potential strategies for m^6^A-targeting therapy.

Target	Name	Effect	Cell type	Tumor	Ref
FTO	FG-2216/IOX3	I	T	melanoma	(94)
	FG-4592/SelleckBio				
	Meclofenamic acid (MA)	I	T		(95)
	Fluorescein	I	T		(96)
	N-CDPCB	I	T		(97)
	CHTB	I	T		(98)
	FB23-2	I	T		(99)
	CS1/CS2	I	T		(100)
ALKBH5	Alk-04	I	T	BC, GBM, LAC, CRC	(82)
	MV1035	I	T		(101)
METTL3-METTL14	small-molecule ligand	A	T	–	(102)
	bisubstrate inhibitor	I	T	CRC, melanoma	(89, 103)
YTHDF1	small molecule, DC vaccine	I	DC	GC, OC, CRC, HCC	(12)
R-2HG	IDH inhibitor	I	T	glioma	(104)

I, inhibit; A, activate; T, tumor; DC, dendritic cell; BC, breast cancer; GBM, glioblastoma; LAC, lung adenocarcinoma; CRC, colorectal cancer; OC, ovarian cancer; HCC, hepatocellular carcinoma.

### Targeting m^6^A Writers

METTL3 and METTL14, as discussed earlier, play an oncogenic role and specifically targeting them can lead to better clinical outcomes. Examples of this include: (1) Depletion of the methyltransferases METLL3 in Tregs which enhances the anti-tumor ability of CD8+ T cells can be used as a strategy alongside immunotherapy for effective treatment (89). (2) Suppressing METTL3 and METTl14 increases CD8+ tumor-infiltrating lymphocytes in addition to the upregulation of IFN-γ, CXCL9, and CXCL10. Although this inhibition alone does not repress tumor growth, it is able to synergize with the immune checkpoint inhibitors such as anti-PD-1 therapy in both CRC and melanoma (13). Unfortunately, selective METTL3/METTL14 inhibitors for cancer therapies are not yet available, but bisubstrate inhibitors are promising small-molecules in that they can be developed to be effective METTL3/METTL14 targeting agents (103, 105).

### Targeting m^6^A Readers

YTHDF1 targeting can be a viable option for cancer therapy, as its elevation promotes cancer growth in different cancers, including GC (85), ovarian cancer (106, 107), CRC (108), and HCC (109). There are different pathways by which YTHDF1 escalates tumor aggressiveness, one of which is through influencing DCs to be less efficient in presenting tumor antigens to T cells. As a result, implementing small molecules or DC vaccines that can suppress YTHDF1 would enhance immunotherapy against cancers (12).

### Targeting m^6^A Erasers

FTO inhibitors can be effective in sensitizing melanoma tumor tissues to anti-PD-1 blockade immunotherapy and at the same time in promoting IFN-γ induced killing, which is also essential for the ICB response (110, 111). In addition, anti-PD-1 immunotherapy effective at the beginning (112), does not have long-lasting effects (110) due to FTO mediated resistance. Therefore, the combination uses of ICB and FTO inhibitors will likely block this resistance in a host with an adaptive immunity presence. m^6^Am^6^AA recent study has also demonstrated that FTO knockdown sensitizes tumors to anti-PD-1 immunotherapy, except that this sensitization effect is not as robust when compared to ALKBH5 depletion (82). Examples of promising FTO inhibitors include the non-steroidal, anti-inflammatory drug, meclofenamic acid (MA), which is a selective FTO inhibitor (95), the ethyl ester form of meclofenamic acid (MA2) (113–115), and FB23-2 (99). In addition, R-2-hydroxyglutarate (R-2HG), an oncometabolite produced by the isocitrate dehydrogenase (IDH) mutations, inhibit FTO activity, leading to the increase of m6A abundance and decreased stability of MYC and CEBPA mRNAs, which subsequently caused growth inhibition, cell cycle arrest and apoptosis of leukemia cells (116).

ALKBH5 has been shown to be responsible for tumor proliferation in different cancers, including breast cancer (117), glioblastoma (118) and LADC (119), so its inhibition can yield good clinical outcomes if implemented. ALKBH5 knockdown has been shown to sensitize melanoma and colon cancer to overcome the resistance for immunotherapy through downregulating prominent cells (Tregs and MDSCs), which immunosuppresses the anti-tumor immunity, while at the same time upregulating DCs (82). One promising ALKBH5 targeting agent is the imidazobenzoxazin-5-thione MV1035 that shows an ALKBH5 inhibition and repression of GBM cell lines (U87-MG cells) through downregulating CD73 expression (101).

### Targeting other m^6^A Regulators

The mechanism by which Wnt/β-catenin signaling promotes tumors includes reducing T cells infiltration in the tumors and subsequently rendering immunotherapeutic approaches ineffective (e.g. anti-PD-L1/anti-CTLA-4 monoclonal antibody therapy) (120). On the other hand, blocking it would prime T cells (e.g., CD8+ T cells) against the tumor and enhance both the DCs’ antigen presentation and immunotherapy effectiveness (121–123). This means that targeting this oncogenic pathway can act as a valuable adjunct to immunotherapy. Thus, several inhibitors that either work through Wnt signals’ membrane transduction blockade as in the case of porcupine inhibitor or through β-catenin phosphorylation promotion as in the case of tankyrase inhibitors were developed and have entered preclinical trials but none of them has yet advanced to clinical use. Some of these inhibitors already show promising results, such as β-catenin inhibitor, PKF115-584, which can synergize with immunotherapies by enhancing DCs activation of T cells; therefore, overcoming the immunosuppression that may be induced by cancers (124).

### m^6^A-Targeting With ICB

**Figure 4:**
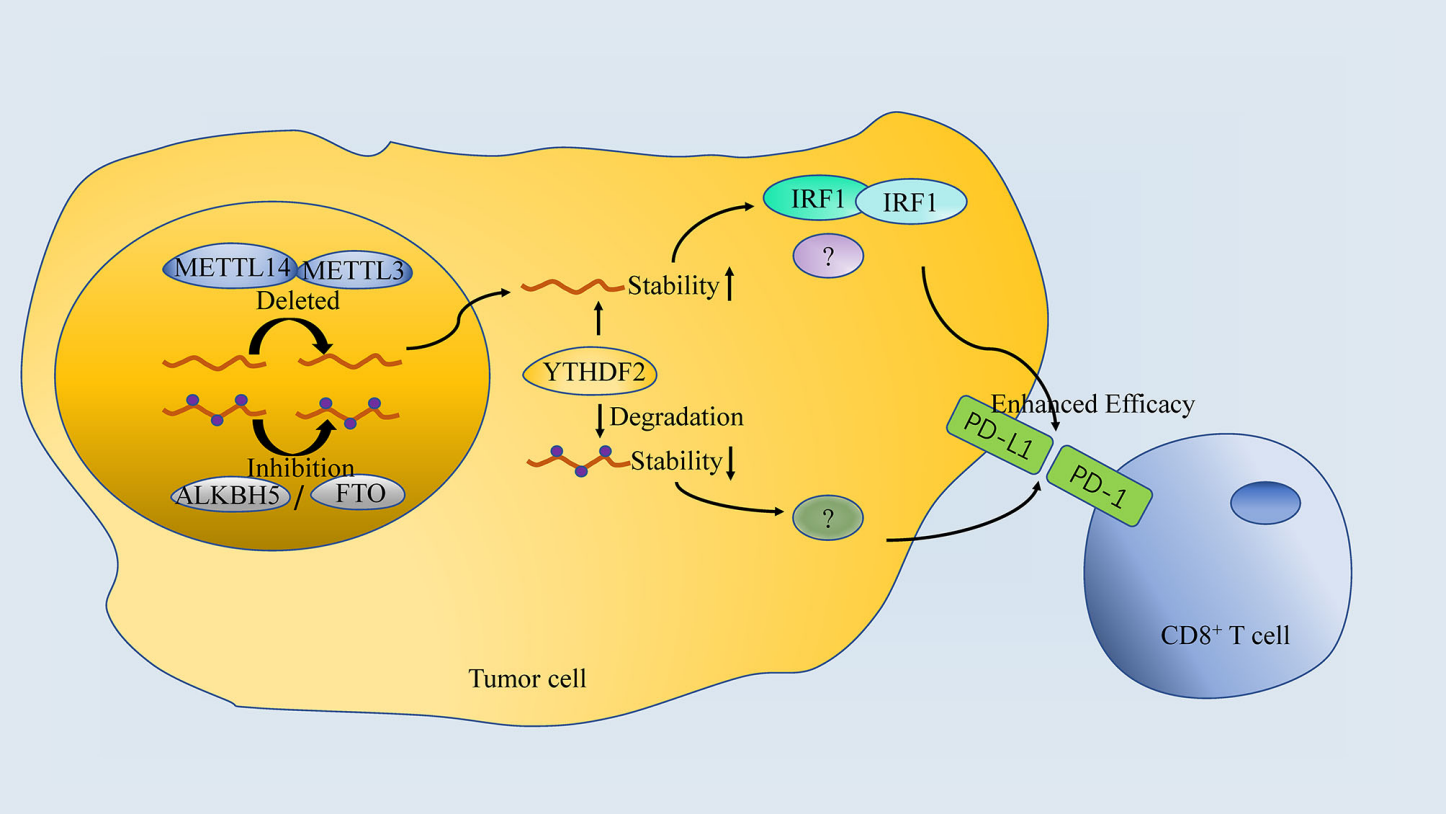
The association between m^6^A regulator and anti-PD-1 treatment. Both Inhibition or Deletion of “writers” or “erasers” enhances the efficacy of anti-PD-1 therapy, which is done by targeting specific RNA in a YTHDF2-dependant manner.

Though ICB (such as anti-PD-1/PD-L1 treatment) have achieved significant clinical benefits in cancer patients receiving ICB, the immunotherapeutic outcomes exhibited individual heterogeneity. Thus, there are two problems that needs to be solved: (1) finding markers to predict the outcomes of immunotherapy; (2) figure out strategy to improve efficacy. Different m^6^A signatures model has been proposed to predict the response of anti-PD-1 immunotherapy (See section”m^6^A is associated with TME and immunotherapy efficacy”). Moreover, m^6^A-targeting treatment synchronizing with anti-PD-1 therapy have already shown improved outcome in preclinical studies (**Figure 4**).

Inhibition of m^6^A mRNA modification by depletion of methyltransferases, Mettl3 and Mettl14, enhanced response to anti-PD-1 treatment in pMMR-MSI-L CRC and melanoma. Mechanistically, Mettl3 or Mettl14 deficiency promoted IFN-γ-Stat1-Irf1 signaling through stabilizing the Stat1 and Irf1 mRNA *via* YTHDF2 (13). However, both FTO and ALKBH5 in tumor cells are proved to decrease response to anti-PD-1 blockade immunotherapy. Knockdown of FTO sensitizes melanoma cells to interferon gamma (IFNγ) and sensitizes melanoma to anti-PD-1 treatment in mice (125). Tumor-intrinsic ALKBH5 inhibited the expansion and cytotoxicity of T cells by sustaining tumor cell PD-L1 expression, which was further confirmed in human intrahepatic cholangiocarcinoma (ICC) specimens (83). Importantly, a small-molecule ALKBH5 inhibitor enhanced the efficacy of cancer immunotherapy in melanoma (82), suggesting that the combination of FTO/ALKBH5 inhibition with anti-PD-1 blockade may reduce the resistance to immunotherapy in melanoma.

Intriguingly, both “writers” inhibition (METTL3 or METTL14) and “erasers” inhibition (FTO or ALKBH5) are proved to enhance the efficacy of anti-PD-1 blockade even in the same cancer (melanoma). It seem contradictory that both of the strategies work, since “writers” and “erasers” are believed to regulate m^6^A modification inversely. The possible explanations for this should include: (1) different targeted mRNA or ncRNA; (2) other potential pathway of “writers” and “erasers”; (3) differences in animal models (gene knockout vs. small molecular inhibitor). In addition, there is insufficient studies regarding the selectivity of target mRNA/ncRNA by “writers”, “readers” and “erasers”, which may explain the question mentioned above. As it is believed that these regulators function by affecting the translation and degradation of specific mRNA/ncRNA, it is reasonable that both “writers” and “erasers” inhibition can synchronize with anti-PD-1 immunotherapy if they act on different targets. However, future researches are needed to verify the hypothesis.

## Conclusions

In this review, we have highlighted the roles of the different players involved in m^6^A modifications, their links to the TME and their potential therapeutic implementation against cancers when they are targeted, and we have particularly emphasized their actions as adjuncts to the various forms of immunotherapy. Most of these therapeutic strategies are still in the preclinical stages and haven’t entered clinical use yet. Nonetheless, these strategies show a promising outlook in impairing cancer progression, especially when bypassing the resistance to immunotherapies (e.g., primary, adaptive, or acquired resistances) through mechanisms such as reversing the immunosuppressive TME by increasing the influx of effector cells. As a consequence, these novel potential targets will be a necessary future component to combine with immunotherapy regimens, when subsequent studies further confirm their efficacy.

## Author Contributions

CQ and OB are major contributors in writing the manuscript. JC and XZ provided guidance throughout the preparation of this manuscript. PL, ZY, and DQ. WR collected and prepared the related papers. JH, HL, AZ, HM, GG, ZQ, MC, and YS reviewed and gave significant advice. All authors contributed to the article and approved the submitted version.

## Funding

This work was supported by the National Natural Science Foundation of China (81873626, 81902592, 82070785), Hunan Natural Science Foundation (2020JJ5884), Hunan Province Key R&D Program (2019SK2202) and Xiangya Hospital Youth Fund (2018Q09).

## Conflict of Interest

The authors declare that the research was conducted in the absence of any commercial or financial relationships that could be construed as a potential conflict of interest.

## Publisher’s Note

All claims expressed in this article are solely those of the authors and do not necessarily represent those of their affiliated organizations, or those of the publisher, the editors and the reviewers. Any product that may be evaluated in this article, or claim that may be made by its manufacturer, is not guaranteed or endorsed by the publisher.
